# Spent Mushroom Substrate Supplemented with *Streptomyces gardneri* MC1-2 Improves Tomato Seedling Growth and Its Establishment in Soil Application

**DOI:** 10.3390/life16101699

**Published:** 2026-10-10

**Authors:** Benyapa Kitwetch, Nilita Mukjang, Yupa Chromkaew, Jenjira Chobyen, Kawiporn Chinachanta, Pharada Rangseekaew, Wasu Pathom-aree

**Affiliations:** 1 Department of Biology, Faculty of Science, Chiang Mai University, Chiang Mai 50200, Thailand; mook_benyapa@hotmail.com (B.K.); nilita.m@cmu.ac.th (N.M.); pharada.rang@cmu.ac.th (P.R.); 2 Center of Excellence in Microbial Diversity and Sustainable Utilization, Faculty of Science, Chiang Mai University, Chiang Mai 50200, Thailand; kawiporn.ch@cmu.ac.th; 3 Department of Plant and Soil Sciences, Faculty of Agriculture, Chiang Mai University, Chiang Mai 50200, Thailand; yupa.c@cmu.ac.th; 4 Department of Entomology and Plant Pathology, Faculty of Agriculture, Chiang Mai University, Chiang Mai 50200, Thailand; jenji.cy@gmail.com

**Keywords:** plant growth-promoting actinobacteria, bioinoculants, spent mushroom substrate, tomato, soil microbiome

## Abstract

Spent mushroom substrate (SMS), a lignocellulosic by-product of mushroom cultivation, represents a promising renewable carrier for microbial bioinoculants as well as agricultural waste valorization. This study evaluated SMS as a carrier for the plant growth-promoting actinobacterium *Streptomyces gardneri* MC1-2 and assessed its effects on tomato growth and soil bacterial communities. Three formulations were prepared: SMS alone (F1), SMS supplemented with 1% (*w*/*w*) carboxymethyl cellulose (CMC) (F2), and talc supplemented with 1% CMC and 1.5% CaCO_3_ (F3). *S. gardneri* MC1-2 exhibited in vitro traits commonly associated with plant-beneficial activity, including indolic compound production, siderophore production, and inorganic phosphate solubilization. During 30 days of storage at 4, 30, and 40 °C, both SMS-based formulations maintained viable populations at a 3 Log CFU reduction, whereas the talc-based formulation lost viability within seven days. Among the formulations, F2 showed the best overall performance and was selected for the pot experiment. Direct inoculation with *S. gardneri* MC1-2 promoted tomato growth comparably to chemical fertilizer, while F2 strongly supported growth in sterile soil, where *S. gardneri* MC1-2 established and dominated the bacterial community. In non-sterile soil, reduced bacterial establishment suggested that competition with native microbiota limited inoculant performance. These findings demonstrate that SMS can serve as an effective carrier for *Streptomyces*-based bioinoculants with potential applications for sustainable crop production.

## 1. Introduction

Modern crop production depends heavily on synthetic fertilizers, the manufacture and overuse of which are associated with high energy consumption, greenhouse-gas emissions, soil degradation, and eutrophication of water bodies. Reducing this dependence without sacrificing yield is a central goal of sustainable agriculture, and microbial inoculants, which contain beneficial microorganisms that improve plant growth and nutrient acquisition, offer an environmentally compatible supplement to, or partial replacement for, chemical inputs [1,2]. Inoculant performance depends on the carrier as much as on the strain, because the carrier must protect the cells, maintain their viability during storage and transport, and deliver them effectively to the plant [1,3].

Meanwhile, expansion of the global mushroom industry generates large quantities of spent mushroom substrate (SMS), the lignocellulosic residue remaining after fruiting-body harvest; approximately five kilograms of SMS are produced per kilogram of fresh mushrooms, and its accumulation and disposal pose growing environmental and economic problems [4,5]. Because SMS retains a substantial fraction of partially degraded lignocellulose, residual organic matter, and mineral nutrients, it has attracted interest as a renewable resource for applications ranging from soil amendment and horticultural growing media to cellulose recovery [4,6,7]. Such uses convert an abundant agricultural waste into a value-added product, consistent with a circular-economy concept.

One promising use of SMS is as a carrier for microbial inoculants, which valorizes the residue while providing a supportive matrix for the introduced organism. Its porous, fibrous structure confers a high water-holding capacity and a large surface area for microbial attachment, while its residual nutrients can sustain cells during storage [3,8]. SMS-based formulations combined with *Bacillus amyloliquefaciens* promoted the growth and chlorophyll content of roselle [8], and a direct comparison of SMS, peat, and biochar as carriers for mineral-solubilizing microorganisms found SMS to give the strongest improvement in plant biomass and soil multifunctionality [9]. SMS therefore offers a renewable alternative to conventional carriers such as peat, which is non-renewable, and inert minerals such as talc.

Members of the genus *Streptomyces* are particularly promising candidates for microbial inoculant formulation. These filamentous actinobacteria produce numerous bioactive metabolites and possess a wide range of plant growth-promoting traits, including auxin (indole-3-acetic acid) production, siderophore-mediated iron acquisition, phosphate solubilization, tolerance of abiotic stress, and suppression of phytopathogens [10,11,12]. *Streptomyces* also reproduce through desiccation-tolerant spores, which resist the physical and chemical stresses of drying, storage, and formulation and make the genus better suited to solid and stable inoculants than most non-spore-forming bacteria [13,14].

Several attempts have been made to develop plant growth-promoting *Streptomyces* into practical biofertilizer products. A talc-based formulation of *S. corchorusii* promoted rice growth and remained viable for at least six months [15], and a kaolin-based formulation of *S. fradiae* NKZ-259 enhanced tomato growth [16]. Growth promotion in these studies varied with the carrier used, indicating that carrier–strain compatibility strongly influences formulation performance [16]. Although SMS has proved suitable as a carrier for other bacteria, its use as a carrier for a plant growth-promoting *Streptomyces* has not, to our knowledge, been reported; actinobacteria have more often been applied to SMS as decomposer inoculum during composting than delivered by it.

The fate of the introduced strain after application to soil is also a consideration in bioinoculant development. An introduced strain must establish and persist within a complex resident soil microbiota, and competition with native communities for nutrients and niche space is a major reason why inoculants that perform well under controlled conditions often fail in the field [17]. Inoculation can also reshape the structure and diversity of the resident community, and the resulting interactions influence both the persistence of the introduced strain and the plant response [18]. Profiling the soil bacterial community after inoculation therefore shows whether an introduced strain establishes and how it interacts with the resident microbiota, yielding information that is important for interpreting inoculant performance and for guiding formulation, but is rarely reported when new carrier materials are evaluated.

This study therefore evaluated SMS as a renewable carrier for the drought-tolerant, cave-derived actinobacterium *S. gardneri* MC1-2. Three carrier formulations were compared for physicochemical properties and for their ability to maintain strain viability during storage at different temperatures. The SMS-based formulation was then assessed for its effects on tomato seed germination, seedling growth, and plant performance in a pot experiment. Its influence on the soil bacterial community after application was also characterized to identify the factors governing inoculant establishment. We aimed to evaluate SMS as a renewable carrier matrix for a plant growth-promoting *Streptomyces* and to assess the survival of the strain in the SMS-based formulation during storage and its effects after application to tomato plants.

## 2. Materials and Methods

### 2.1. Plant Growth-Promoting Properties of Streptomyces gardneri MC1-2

*S. gardneri* MC1-2 was isolated from the cotton swab samples from walls within Chiang Dao cave, Chiang Mai province, Thailand, using D-medium and incubated at room temperature (28 ± 2 °C) for two weeks [19]. The purified isolate was routinely cultivated on International *Streptomyces* Project medium 2 (ISP2) agar at room temperature as a working culture and preserved as a mixture of spore and cell suspension in 20% (*v*/*v*) glycerol at −20 °C for long-term storage. Isolate MC1-2 was identified by 16S rRNA gene sequence analysis. Genomic DNA of strain MC1-2 was extracted, and the 16S rRNA gene was amplified using primers 27F and 1492R with MyTaq™ Master Mix (Bioline, London, UK). The PCR product was purified and subjected to Sanger sequencing by U2Bio Sequencing Service, Thailand. The obtained sequences were aligned and assembled using GeneStudio™ Professional and BioEdit, and the resulting sequence was compared with reference sequences using the BLAST analysis option available on the EzBioCloud web server (http://www.ezbiocloud.net/taxonomy, accessed on 26 March 2022). The 16S rRNA gene sequence of strain MC1-2 was deposited in GenBank under accession number LC703263.

Total indolic compound production, siderophore production, and phosphate solubilization by *S. gardneri* MC1-2 were evaluated using standard methods. Total indolic compound production was quantified using the colorimetric method described in Rangseekaew et al. (2021) [20] with minor modifications. Briefly, *S. gardneri* MC1-2 was cultured in ISP2 broth supplemented with 2 mg mL^−1^ L-tryptophan and incubated at 110 rpm in the dark for 7 days. The culture supernatant was mixed with Salkowski’s reagent [21], incubated in the dark for 30 min, and the absorbance was measured at 530 nm using a microplate reader (SpectraMax iD3, San Jose, CA, USA). Total indole concentration was calculated from a standard curve and expressed as µg mL^−1^.

Siderophore production by *S. gardneri* MC1-2 was qualitatively screened using Chrome Azurol S (CAS) agar and incubated at room temperature (28 ± 2 °C) in the dark for 7 days. The formation of a yellow halo indicated siderophore production. Quantitative siderophore production was determined after shaking cultivation in King’s B broth at 110 rpm for 7 days at room temperature. Culture supernatant was collected for the quantification of hydroxamate- and catecholate-type siderophores. Hydroxamate-type siderophores were quantified using the ferric perchlorate assay [22]. Briefly, the supernatant was mixed with ferric perchlorate solution, incubated at room temperature for 5 min, and the absorbance was measured at 480 nm using a microplate reader. Siderophore concentration was calculated from a deferoxamine mesylate standard curve. Catecholate-type siderophores were quantified using the Arnow assay [23]. The collected supernatant was sequentially mixed with 0.5 M hydrochloric acid, nitrite-molybdate reagent, and sodium hydroxide, followed by incubation at room temperature for 5 min. Absorbance was measured at 500 nm using a microplate reader, and catecholate-type siderophore concentration was calculated from a 2,3-dihydroxybenzoic acid standard curve.

Phosphate solubilization was assayed on Pikovskaya’s (PVK) agar supplemented with 0.5% (*w*/*v*) tricalcium phosphate [Ca_3_(PO_4_)_2_] as described previously [20]. The formation of a clear halo indicated phosphate solubilization. For quantitative analysis, *S. gardneri* MC1-2 was cultured in Pikovskaya’s broth supplemented with 0.5% (*w*/*v*) Ca_3_(PO_4_)_2_ and incubated on a shaker at 110 rpm for 7 days at room temperature. The culture supernatant was mixed with 10% (*v*/*v*) trichloroacetic acid, followed by the addition of color reagent (6 N H_2_SO_4_, 2.5% (*w*/*v*) ammonium molybdate, 10% (*w*/*v*) ascorbic acid, and distilled water; 1:1:1:2, *v*/*v*/*v*/*v*), then incubated at room temperature for 15 min, and the absorbance was measured at 820 nm using a microplate reader. Soluble phosphorus concentration was determined from a KH_2_PO_4_ standard curve [20].

Drought tolerance ability of *S. gardneri* MC1-2 was tested using the streak-plate technique on 10% tryptic soy agar supplemented with 0 gL^−1^, 85 gL^−1^, 285 gL^−1^, 405 gL^−1^, 520 gL^−1^ and 660 gL^−1^ of sorbitol to adjust the a_w_ value of the media to simulate water stress at 37 °C for 7 days. The final aw values of the media were 0.998, 0.986, 0.957, 0.919, 0.897, and 0.844, respectively. Actinobacteria that grew at an aw of 0.919 were considered drought tolerant [19,24].

### 2.2. Bacterial Cultivation and Bioinoculant Preparation

For spore production, *S. gardneri* MC1-2 was cultured on mannitol soybean (MSB) agar containing 20 g/L of mannitol, 20 g/L of soybean powder, and 18 g/L of agar at 28 °C for 14 days. Fully developed spores were collected and suspended in sterile distilled water to adjust to a final concentration of 10^8^ CFU/ml (OD_600_ = 1.00). The spent mushroom substrate of branched oyster mushroom (*Pleurotus cornucopiae*) was obtained from Ban-Hed Su-Pha-Wat Farm located in Saraphi District, Chiang Mai, Thailand. The SMS contained 28.34% cellulose, 18.09% hemicellulose, and 37.59% lignin [7]. Before use, the dried SMS was sieved to a particle size of approximately 5–10 mm, autoclaved at 121 °C for 15 min on two consecutive days and dried at 60 °C overnight. The bioinoculant was prepared using sterile spent mushroom substrate (F1) and sterile spent mushroom substrate (SMS) supplemented with 1% (*w*/*w*) carboxymethyl cellulose (CMC) (Sigma-Aldrich, St. Louis, MO, USA) (F2). A talcum powder-based formulation (talcum powder + 1% (*w*/*w*) CMC + 1.5% (*w*/*w*) CaCO_3_ (Loba Chemie, Mumbai, India), F3) was also prepared for comparison [25]. All formulations were inoculated with a spore suspension of *S. gardneri* MC1-2 to a final concentration of 10^7^ CFU/g. For the stability trial, 5 g of each formulation was dispensed into sterile aluminum foil bags (7 × 10 cm), heat-sealed and stored aerobically. All bags were stored in the dark at 4 °C, 30 °C and 40 °C for 30 days. Viable counts were determined by serial dilution and spread-plating on ISP2 agar and expressed as CFU/g dry weight (n = 3) on day 0, 7, 15 and 30 at each storage temperature.

### 2.3. Germination and Early Seedling Growth Assays

Plum tomato (*Lycopersicon esculentum* Mill) seeds were obtained from Chia Tai Co., Ltd., Thailand. Seeds were surface sterilized by immersion in 70% (*v*/*v*) ethanol for 3 min, followed by incubation in 5% (*v*/*v*) NaClO for 5 min with shaking. The seeds were then washed six times with sterile distilled water [26]. In addition, three sterilized seeds were randomly placed on ISP2 or nutrient agar and incubated at 37 °C for 48 h to confirm surface sterility. Sterile peat moss was used as the substrate for the tomato seed germination assay. Each bioinoculant was mixed with peat moss at a ratio of 1:10 (*w*/*w*), while sterile distilled water served as a negative control. The experiment consisted of eight treatments: the three bioinoculant formulations (F1–F3), each with and without *S. gardneri* MC1-2, together with a positive control (peat moss + *S. gardneri* MC1-2 spore suspension) and a negative control (peat moss + sterile distilled water). A total of 40 surface-sterilized tomato seeds from each treatment were sown and incubated under LED artificial sunlight at room temperature. After 7 days, germination percentage was determined. Seedling growth was subsequently evaluated by measuring shoot and root length, fresh and dry biomass, and chlorophyll and carotenoid contents [27].

### 2.4. Plant Growth Promotion of SMS-Based Bioinoculant in Pot Experiment

The SMS-based bioinoculant (F2) was further evaluated for its growth-promoting effect on plum tomato (*Lycopersicon esculentum* Mill) under pot conditions. Sterile tomato seeds were sown in 2.5-inch black plastic pots (6 cm top diameter, 4.6 cm base diameter, and 5.6 cm height) with the following treatments (20 pots per treatment):

T1: non-sterile soil as a control;

T2: non-sterile soil and SMS;

T3: non-sterile soil and chemical fertilizer (N: P: K, 16: 16: 16);

T4: non-sterile soil and *S. gardneri* MC1-2;

T5: non-sterile soil and SMS-based bioinoculant F2;

T6: sterile soil and SMS-based bioinoculant F2.

The composition of each treatment is shown in Table 1. The pots were kept in the dark for 24 h to stimulate seed germination. Thereafter, each pot received 5 mL of sterile distilled water twice daily, in the morning and in the late afternoon over the 45-day trial. Subsequently, the tomato pots were placed under a 12 h light/12 h dark photoperiod. After 45 days of cultivation, the morphological parameters (n = 6), including shoot length, root length, fresh weight and dry weight, were measured. Chlorophyll content was determined using the standard method [27]. Briefly, 1 g of fresh tomato leaves was homogenized with 80% (*v*/*v*) acetone, and the homogenate was centrifuged at 13,000 rpm for 10 min. The supernatant was collected, and absorbance was measured at 480, 645 and 663 nm using a spectrophotometer (n = 9). The total chlorophyll content was calculated using the following formulas:

Total chlorophyll (mg/g) = 20.2 × OD_645_ + 8.02 × OD_663_ × V/(1000 × W)

Carotenoids (mg/g) = 4.695 × OD_480_ − 0.268 × V/(1000 × W)

where V is the volume of 80% acetone (mL), and W is the fresh weight of tomato leaves (g).

Total soluble sugar content was determined according to Dubois [20,28]. Briefly, 1 g of fresh tomato leaves was homogenized with 80% (*v*/*v*) ethanol and incubated at 75 °C for 15 min. The supernatant was collected by centrifugation at 13,000 rpm for 10 min. Then, 250 μL of supernatant was mixed with 250 μL of 80% (*v*/*v*) ethanol, 2.5 mL of H_2_SO_4_, and 500 μL of 5% (*v*/*v*) phenol, respectively. The mixture was mixed well and incubated at room temperature for 20 min. The absorbance of the mixture was measured spectrophotometrically at 490 nm (n = 3). Total soluble sugar content was calculated from a standard curve prepared with known concentrations of glucose and expressed as mg/g fresh weight.

### 2.5. Colonization Assay

To confirm the colonization of *S. gardneri* MC1-2 in the rhizosphere soil and root of the tomato plant. The growth substrate supplemented with *S. gardneri* MC1-2 from treatments T4, T5, and T6 was collected in a sterile plastic bag at the end of the pot experiment and kept at 4 °C for analysis. Briefly, 10 g of the growth substrate was mixed with 90 mL of 0.85% (*w*/*v*) NaCl and serially diluted 10-fold to 10^−6^. Each dilution was spread on ISP2 agar plates supplemented with 25 μg/mL nalidixic acid and 25 μg/mL ketoconazole in triplicate and incubated at 28 °C ± 2 °C for 14 days. The presumptive colony of *S. gardneri* MC1-2 was counted and expressed as CFU per gram of fresh substrate. The tomato root was harvested at 45 DAT and cleaned with sterile distilled water 3 times. The root was stored in a sterile bottle containing TE buffer and kept at 4 °C for analysis. Briefly, 10 g of roots were sterilized by soaking in 2% (*v*/*v*) NaOCl for 3 min and washing 4 times in sterile distilled water [29]. The sterile root was crushed with 90 mL of 0.85% (*w*/*v*) NaCl and serially diluted 10-fold to 10^−6^. Each dilution was spread on ISP2 agar plates supplemented with 25 μg/mL nalidixic acid and 25 μg/mL ketoconazole in triplicate and incubated at 28 °C ± 2 °C for 14 days. The presumptive count of *S. gardneri* MC1-2 was expressed as CFU/g root fresh weight.

### 2.6. Soil Bacterial Community Analysis

DNA was extracted from soil samples of each treatment using the Quick-DNA™ Fecal/Soil Microbe Miniprep Kit (Zymo Research, Irvine, CA, USA) from six treatments (T1–T6) sampled at two planting times, which are day 0 and day 45, with three biological replicates per treatment × time combination (n  =  36 samples total). PCR amplification of the bacterial 16S rRNA gene was performed to confirm the quality of the extracted DNA and ensure the absence of PCR inhibitors using 515F and 806R primers. The extracted DNA was subjected to amplicon metagenomic sequencing (MiSeq platform, Illumina, San Diego, CA, USA) targeting the V3-V4 regions of the bacterial 16S rRNA gene. Demultiplexed paired-end sequence reads were processed in R using the DADA2 pipeline following Callahan et al. [30]. Sequence quality profiles were first inspected, after which reads were filtered and trimmed based on their quality profiles. Reads containing ambiguous bases were removed, and low-quality reads were filtered. Error rates were then learned independently from the filtered forward and reverse reads using the DADA2 error model. Identical reads were dereplicated, and amplicon sequence variants (ASVs) were inferred from the dereplicated reads using the DADA algorithm. Denoised forward and reverse reads were subsequently merged on the basis of their overlapping regions, and an ASV sequence table was constructed. Chimeric sequences were identified and removed de novo using the consensus method implemented in DADA2.

Taxonomic assignment of the non-chimeric ASVs was performed against the SILVA reference database using the taxonomic classification procedure implemented in DADA2. The resulting ASV abundance table and taxonomic assignments were imported into the phyloseq package for downstream microbiome analyses in R (R Core Team, Vienna, Austria). For community-level analyses, ASV counts were converted to relative abundance within each sample prior to comparative analyses rather than rarefying the sequence data. Bray–Curtis dissimilarities were calculated from relative-abundance ASV profiles for all 36 samples. Differences in bacterial community composition among treatments were evaluated by permutational multivariate analysis of variance (PERMANOVA) using the adonis function in the vegan package. The effects of treatment and sampling time on bacterial diversity were evaluated using analysis of variance (ANOVA). Taxonomic relative abundances were summarized at the relevant taxonomic levels for comparison among treatments and sampling times. Raw sequencing reads generated in this study were deposited in the NCBI Sequence Read Archive under BioProject accession (PRJNA1509567).

### 2.7. Statistical Analysis

The experiments were conducted in a completely randomized design. The number of biological replicates was as follows: bioinoculant characterization (n = 3), seedling growth parameters (n = 3), pot-experiment morphological parameters (n = 6), chlorophyll content (n = 9), and total soluble sugar content (n = 3). Data are presented as mean values with standard deviation. Differences among treatment means were evaluated by one-way analysis of variance (ANOVA), followed by Tukey’s multiple range test as the post-hoc procedure, with significance accepted at *p* < 0.05. All statistical analyses were performed using IBM SPSS Statistics version 22.0 (IBM Corp., Armonk, NY, USA).

## 3. Results

### 3.1. Plant Growth-Promoting Properties of S. gardneri MC1-2

The 16S rRNA gene sequence of strain MC1-2 was 1406 bp in length and showed 99.5% sequence similarity to *Streptomyces gardneri* NBRC 12865, supporting its identification as *S. gardneri* MC1-2. *S. gardneri* MC1-2 was capable of multiple plant growth-promoting (PGP) characteristics. The strain produced 7.98 ± 0.78 µg mL^−1^ of total indolic compounds. Siderophore production was indicated by the formation of a 1.53 ± 0.05 cm yellow halo on chrome azurol S (CAS) agar. Subsequent assays indicated that *S. gardneri* MC1-2 produced hydroxamate-type siderophores, at 486.67 ± 46.65 µmol L^−1^, together with a smaller quantity of catecholate-type siderophores (33.16 ± 7.08 µmol L^−1^). The strain also exhibited an ability to solubilize inorganic phosphate by forming a 0.60 ± 0.00 cm clear zone on Pikovskaya’s agar and released 83.33 ± 3.44 µg mL^−1^ soluble phosphorus in liquid medium containing tricalcium phosphate. A decrease in the pH of the culture broth from 7.00 to 5.22 ± 0.01 occurred during phosphate solubilization. In addition to these PGP traits, MC1-2 was able to grow under reduced water availability at a water activity (a_w_) of 0.919, indicating tolerance to low-moisture conditions.

### 3.2. Physicochemical Properties of SMS-Based Bioinoculant and Survival of S. gardneri MC1-2

**Figure 1:**
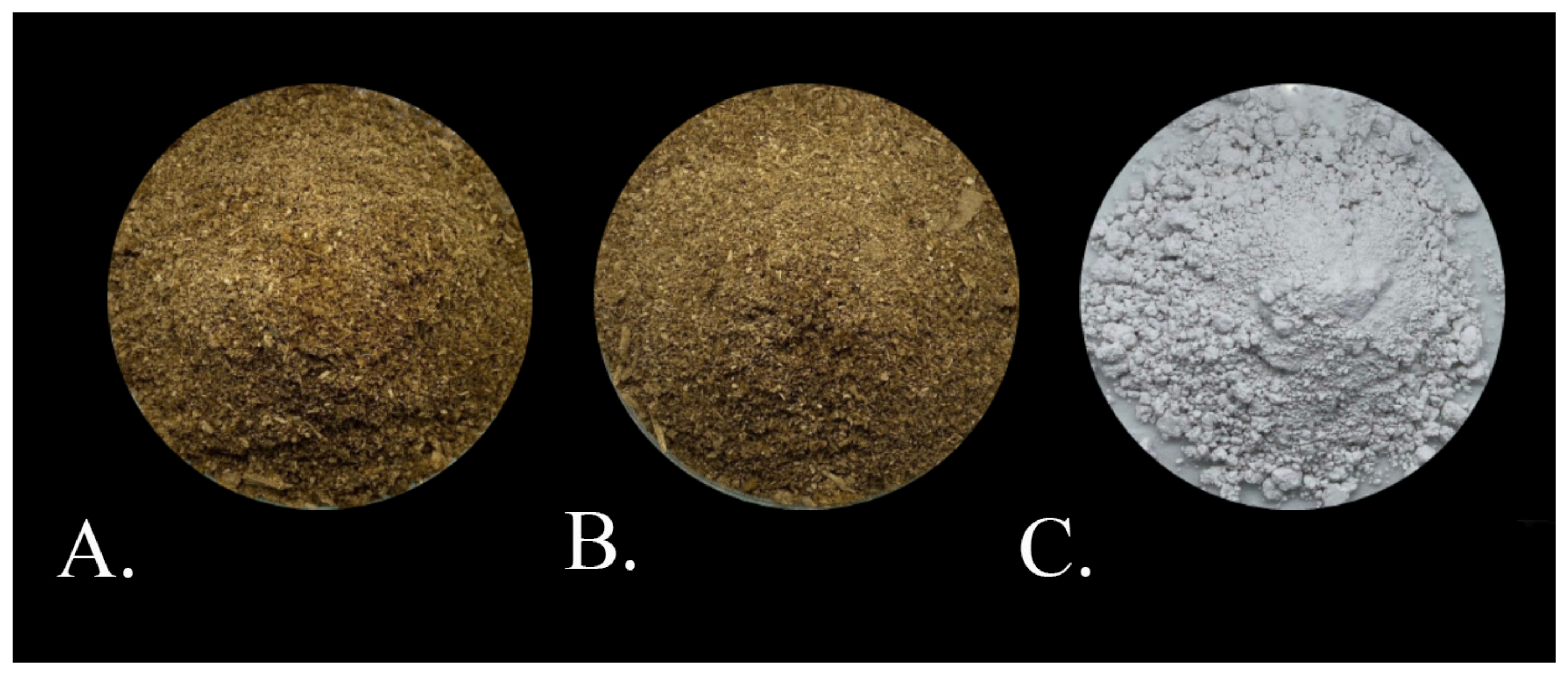
Physical appearance of bioinoculant formulations: (**A**) F1 (SMS); (**B**) F2 (SMS + 1% (*w*/*w*) CMC); and (**C**) F3 (Talcum powder + 1% (*w*/*w*) CMC + 1.5% (*w*/*w*) CaCO_3_).

Carrier composition clearly affected the physicochemical properties of the prepared bioinoculants. The SMS-based materials, SMS (F1) and SMS supplemented with 1% (*w*/*v*) CMC (F2), showed the brown color of the original substrate, while the talc-based formulation F3 was gray, as expected for a mineral talcum powder (Figure 1). The SMS contained 28.34% cellulose, 18.09% hemicellulose, and 37.59% lignin, indicating that rich lignocellulose remained after mushroom cultivation.

The SMS-based formulations F1 and F2 were mildly acidic, whereas F3 exhibited a near-neutral pH (Table 2). The pH of the SMS-based formulations remained weakly acidic throughout the 30-day storage at 4 °C, 30 °C, and 40 °C, whereas the pH of F3 increased toward alkaline values (Table 3). Carrier composition also strongly influenced electrical conductivity (EC), as observed from substantial differences in EC values between formulations. F2 showed EC values of 2290 µS cm^−1^, closely similar to that of SMS alone (2263 µS cm^−1^), whereas F3 exhibited a considerably lower EC value of 427 µS cm^−1^ (Table 4).

During storage at 4 °C, 30 °C, and 40 °C, the physicochemical characteristics of the formulations and survival of *S. gardneri* MC1-2 changed substantially, with distinct responses according to carrier composition, temperature, and storage duration (Table 3, Table 4 and Table 5). At day 0, initial populations of *S. gardneri* MC1-2 were 5.35 ± 1.48 × 10^7^, 3.90 ± 1.13 × 10^7^, and 3.70 ± 0.42 × 10^7^ CFU g^−1^ in F1, F2, and F3, respectively (Table 5). During the first 7 days, the EC values increased substantially in both SMS formulations at all temperatures. In F1, EC increased by approximately 31–34%, reaching 2970–3020 µS cm^−1^, whereas F2 showed increases of approximately 33–40%. However, these increases were not accompanied by an increase in *S. gardneri* MC1-2. Populations in F1 remained between 3.70 × 10^7^ and 4.60 × 10^7^ CFU g^−1^, while those in F2 ranged from 2.15 × 10^7^ to 3.50 × 10^7^ CFU g^−1^.

In contrast to the SMS-based formulations, *S. gardneri* MC1-2 was not detected in F3 after 7 days at any tested temperature. This loss of viability corresponded with a reduction in the EC values from 427 µS cm^−1^ to 279–316 µS cm^−1^. A pronounced loss of *S. gardneri* MC1-2 viability occurred between days 7 and 15 in both SMS-based formulations (Table 5). Populations of *S. gardneri* MC1-2 in F1 declined to 3.23 × 10^6^, 3.47 × 10^6^, and 4.40 × 10^6^ CFU g^−1^ at 4 °C, 30 °C, and 40 °C, respectively. Similarly, populations of *S. gardneri* MC1-2 in F2 declined to 3.10 × 10^5^, 6.47 × 10^5^, and 5.10 × 10^6^ CFU g^−1^. During the same period, EC values generally stabilized or declined, although values remained substantially higher than their respective initial levels. After 30 days, the viable population of *S. gardneri* MC1-2 further declined in both SMS-based formulations but remained detectable at all temperatures (Table 5). F1 retained 3.84 × 10^5^ CFU g^−1^ at 4 °C, 2.67 × 10^4^ CFU g^−1^ at 30 °C, and 3.70 × 10^4^ CFU g^−1^ at 40 °C. Corresponding populations of *S. gardneri* MC1-2 in F2 were 8.60 × 10^4^, 5.03 × 10^4^, and 5.30 × 10^4^ CFU g^−1^, respectively.

Despite the substantial reduction in viable cells, the EC values remained elevated. For F1, the EC values of 2790, 2810, and 2720 µS cm^−1^ were recorded at 4 °C, 30 °C, and 40 °C, representing an increase of approximately 20–24% relative to day 0 (Table 4). F2 exhibited even greater net increases, reaching 3100, 2990, and 3350 µS cm^−1^, equivalent to approximately 31–46% increases. The highest increase was observed in F2 stored at 40 °C, where the EC value increased by 1060 µS cm^−1^ (+46.3%) despite a reduction in the viable population of *S. gardneri* MC1-2 from 3.90 × 10^7^ to 5.30 × 10^4^ CFU g^−1^. In contrast, F3 exhibited a complete loss of detectable *S. gardneri* MC1-2 population after 7 days and a reduction in EC values, reaching 321–341 µS cm^−1^ by day 30. Although both SMS-based formulations F1 and F2 displayed similar initial EC values, the net EC increase in F2 after 30 days exceeded that of F1 by 283, 153, and 603 µS cm^−1^ at 4 °C, 30 °C, and 40 °C, respectively.

### 3.3. Germination and Early Seedling Growth

**Figure 2:**
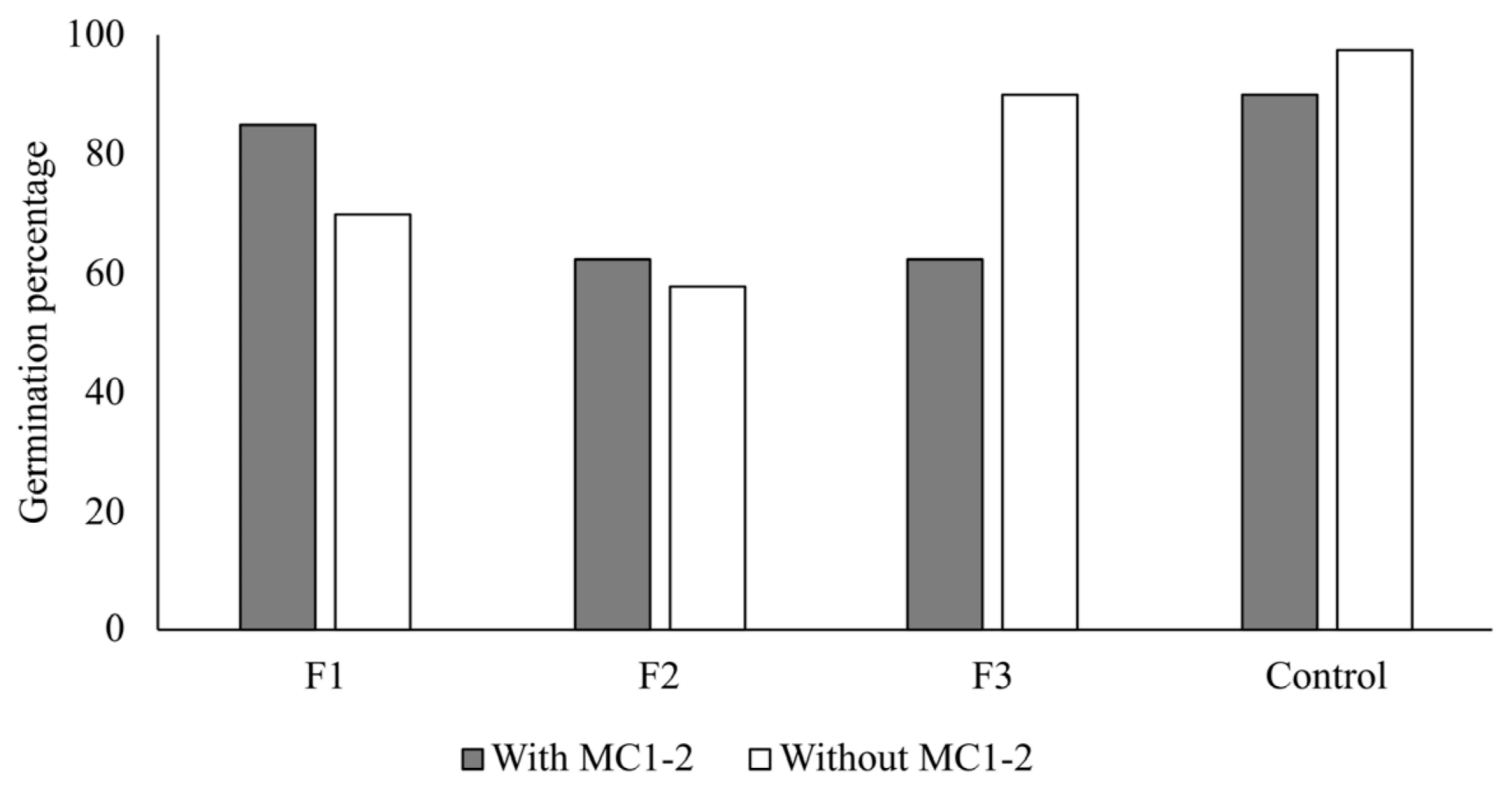
The germination percentages of tomato seeds grown in bioinoculant formulations (F1–F3) with and without inoculation of *S. gardneri* MC1-2. The control treatment consisted of peat moss with (positive control) and without (negative control) MC1-2 inoculation.

The highest germination percentages were observed in the negative and positive controls, indicating that peat moss provided favorable conditions for tomato seed germination and that direct application of the *S. gardneri* MC1-2 spore suspension did not adversely affect germination (Figure 2). The effect of *S. gardneri* MC1-2 incorporation differed among carrier formulations. In both SMS-based formulations, inoculation with *S. gardneri* MC1-2 numerically increased germination relative to the corresponding uninoculated carriers, with the greatest difference observed in F1.

Nevertheless, germination in the SMS-based treatments generally remained lower than that of the peat-based controls, indicating that the beneficial effect associated with *S. gardneri* MC1-2 did not completely overcome carrier-related constraints. In contrast to the SMS-based formulations, the talc-based F3 formulation with *S. gardneri* MC1-2 resulted in lower germination than the corresponding uninoculated carrier. After 21 days of cultivation, tomato seedling growth differed among the carrier–inoculant combinations (Table 6). The SMS-based F2 formulation supplemented with *S. gardneri* MC1-2 consistently produced the highest mean values for fresh weight, dry weight, shoot length, and root length (Table 6). Although F1 and F2 showed similar survival patterns during storage, F2 was selected for further evaluation primarily based on its consistently favorable plant-growth performance across the evaluated seedling growth parameters.

### 3.4. Effect of SMS-Based Bioinoculant F2 on Tomato Growth and Physiological Characteristics

**Figure 3:**
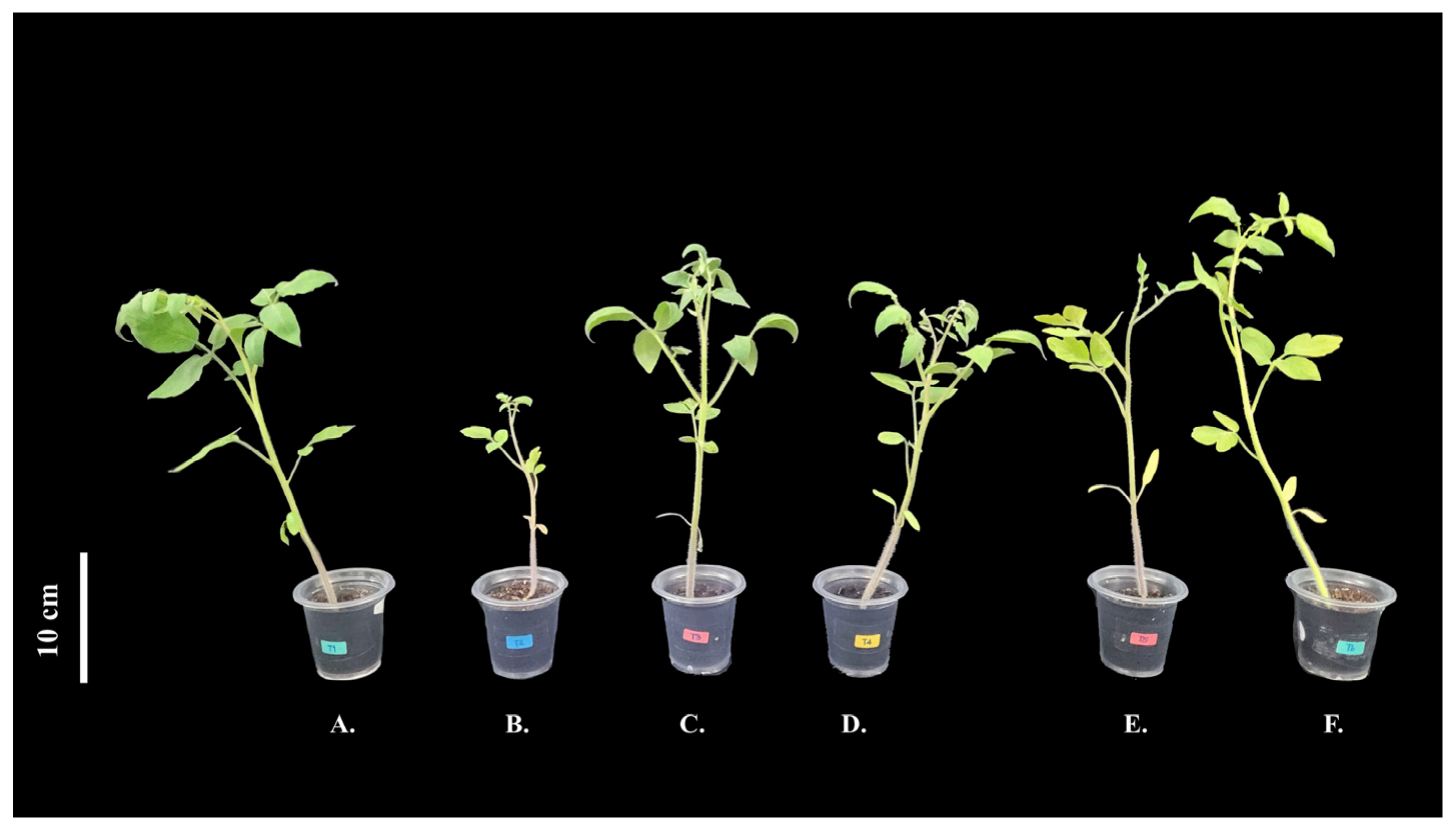
Growth performance of tomato plants under different soil amendment and bioinoculant treatments at the end of the pot experiment. (**A**) T1, non-sterile soil control; (**B**) T2, non-sterile soil + SMS; (**C**) T3, non-sterile soil + chemical fertilizer (16:16:16); (**D**) T4, non-sterile soil + *S. gardneri* MC1-2 spore suspension; (**E**) T5, non-sterile soil + F2; and (**F**) T6, sterile soil + F2.

**Figure 4:**
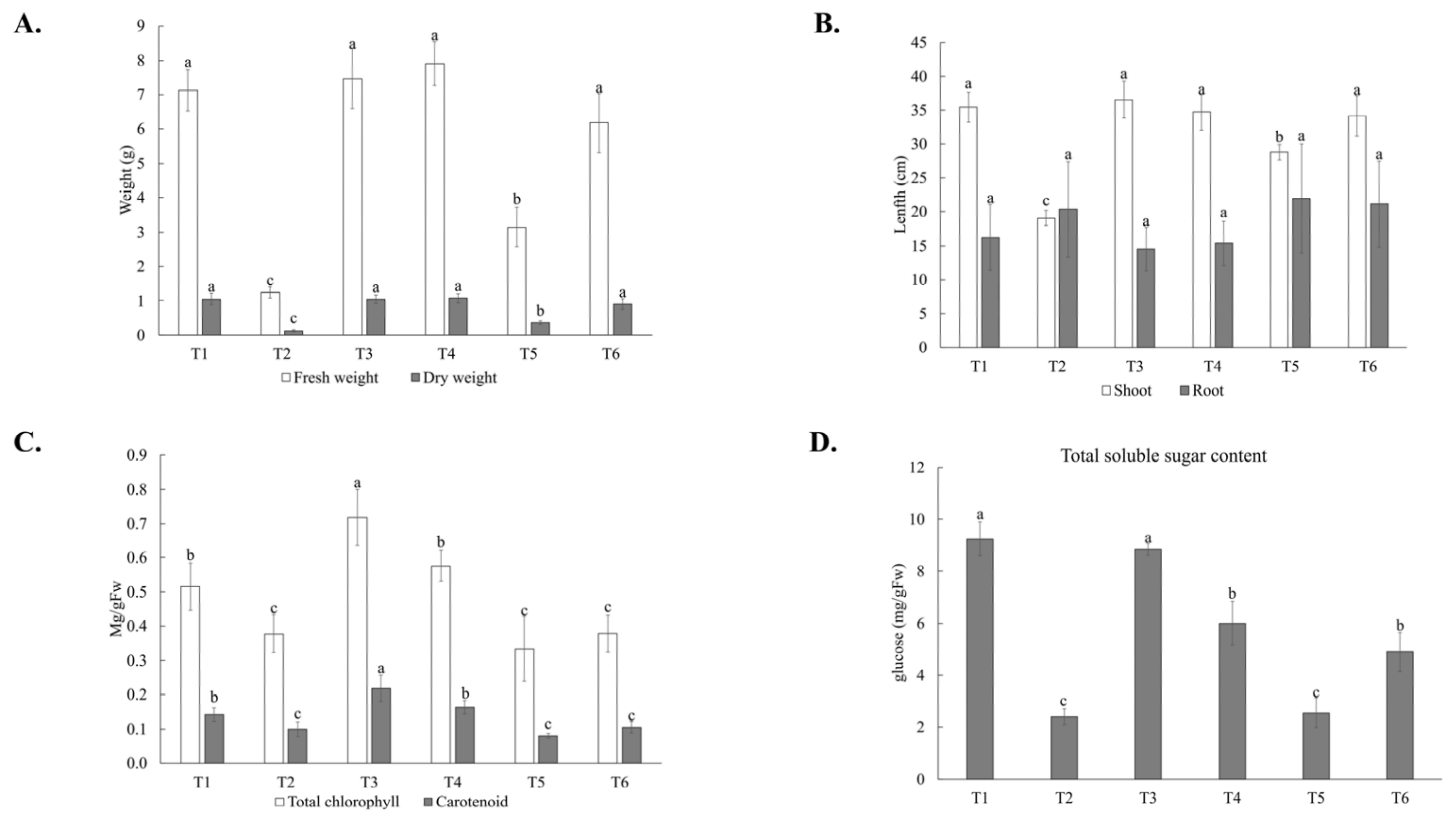
Effects of different soil amendments and bioinoculant treatments on (**A**) fresh and dry weight; (**B**) shoot and root length; (**C**) total chlorophyll and carotenoid contents; and (**D**) total soluble sugar content. T1: non-sterile soil, T2: non-sterile soil and SMS, T3: non-sterile soil and chemical fertilizer, T4: non-sterile soil and spore suspension of *S. gardneri* MC1-2, T5: non-sterile soil and bioinoculant F2 (*S. gardneri* MC1-2), and T6: sterile soil and bioinoculant F2 (*S. gardneri* MC1-2). The different superscript letters (a, b, c) within the same column indicate significant differences between treatments according to Tukey’s multiple range test, *p* < 0.05.

The performance of SMS-based F2 formulation on tomato growth was evaluated in pot culture for 45 days. Six treatments were compared: non-sterile soil control (T1), non-sterile soil and SMS (T2), non-sterile soil and chemical fertilizer (16:16:16) (T3), non-sterile soil and *S. gardneri* MC1-2 (T4), non-sterile soil and SMS-based bioinoculant F2 (T5), and sterile soil and SMS-based bioinoculant F2 (T6) (Figure 3 and Figure 4). Direct inoculation with *S. gardneri* MC1-2 (T4) produced fresh weight (7.91 g), dry weight (1.07 g), and shoot length (34.68 cm) that were statistically comparable to those obtained with the chemical fertilizer treatment (T3) and the non-sterile soil control (T1). In contrast, the SMS treatment without *S. gardneri* MC1-2 (T2) resulted in the lowest biomass and shoot growth, while F2 applied to non-sterile soil (T5) showed intermediate performance. Interestingly, F2 applied to sterile soil (T6) showed tomato growth statistically comparable to chemical fertilizer treatment (T3) and direct inoculation with *S. gardneri* MC1-2 (T4), which suggests that the performance of *S. gardneri* MC1-2 was not affected by the formulation procedure. Root length was similar across all treatments, indicating that treatments affected shoot growth and biomass accumulation more than root elongation.

Total chlorophyll and carotenoid contents followed the same trend as biomass accumulation. Chemical fertilizer (T3) gave the highest chlorophyll, carotenoid, and total soluble sugar contents, whereas the SMS-containing treatment showed low photosynthesis pigment contents. Since chlorophyll and carotenoids are responsible for light harvesting and photoprotection, the higher photosynthesis pigment contents corresponded to the higher total soluble sugar content. In contrast, F2 applied to non-sterile soil (T5) produced markedly lower biomass than the direct *S. gardneri* MC1-2 treatment, whereas F2 in sterile soil (T6) supported growth comparable to T3 and T4. The highest number of re-isolated putative *S. gardneri* MC1-2 population was recovered from sterile soil treatment T6 (3.33 × 10^6^ CFU/g), with lower populations in T5 (6.3 × 10^5^ CFU/g) and T4 (5.6 × 10^5^ CFU/g). *S. gardneri* MC1-2 was not detected in surface-sterilized roots, indicating that it preferably colonized the substrate and rhizosphere rather than the root interior.

### 3.5. Soil Bacterial Community Analysis

**Figure 5:**
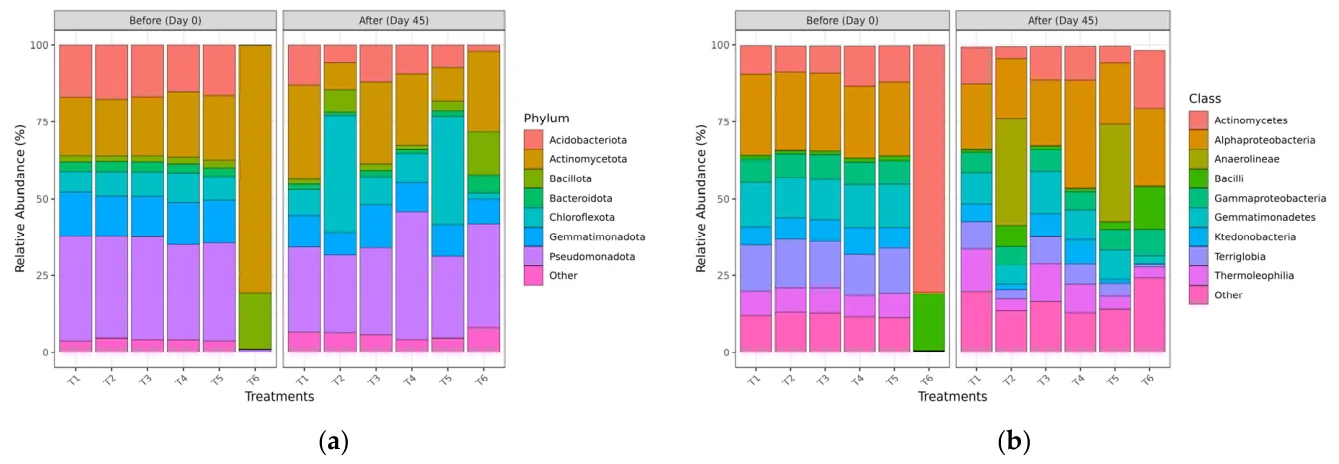
Bacterial communities at the phylum (**a**) and class level (**b**) in the soil of different treatments, shown separately for Day 0 (Before planting) and Day 45 (After harvest). Bars show the mean relative abundance of each replicate group (n  =  3. T1: non-sterile soil, T2: non-sterile soil and SMS, T3: non-sterile soil and chemical fertilizer, T4: non-sterile soil and spore suspension of *S. gardneri* MC1-2, T5: non-sterile soil and bioinoculant F2 (*S. gardneri* MC1-2), and T6: sterile soil and bioinoculant F2 (*S. gardneri* MC1-2). Composition was compared with a sample-level PERMANOVA on Bray–Curtis dissimilarities (see Results for the full statistics).

**Figure 6:**
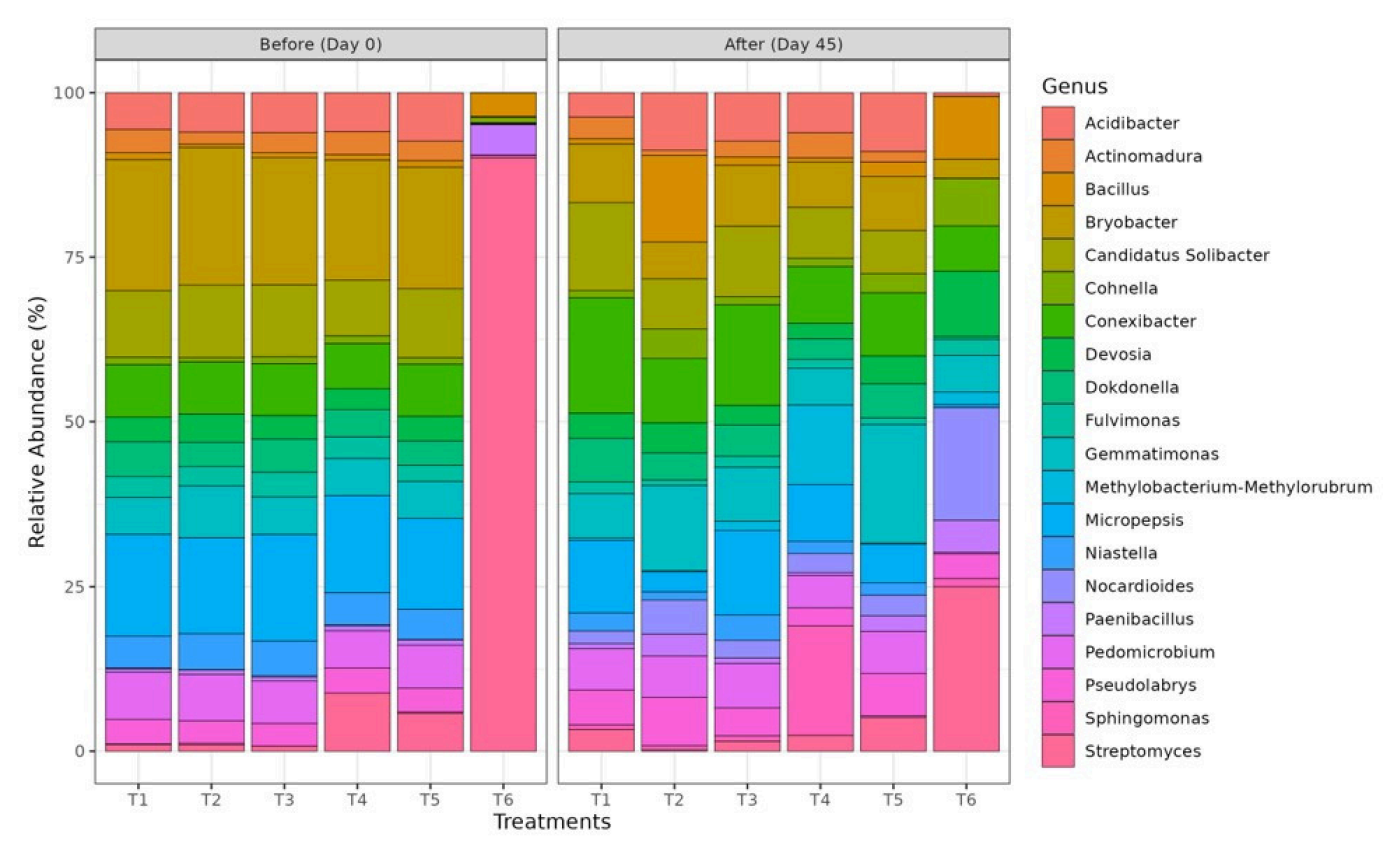
Composition of the top 20 bacterial genera at Day 0 (before planting) and Day 45 (after harvest). For each sample, the relative abundances of the selected top 20 genera were renormalized so that their sum equaled 100%, and the resulting proportions were averaged within each treatment and sampling time. Thus, percentages represent the relative distribution among the top 20 genera and not their abundance as a percentage of the total bacterial community. Genera outside the top 20 and reads not classified at the genus level are not represented. T1: non-sterile soil; T2: non-sterile soil and SMS; T3: non-sterile soil and chemical fertilizer; T4: non-sterile soil and spore suspension of *S. gardneri* MC1-2; T5: non-sterile soil and bioinoculant F2 (*S. gardneri* MC1-2); and T6: sterile soil and bioinoculant F2 (*S. gardneri* MC1-2).

The relative abundance of bacterial taxa in soil under six different treatments (T1 to T6) is shown in Figure 5. A sample-level PERMANOVA on Bray–Curtis dissimilarities (36 samples, 999 permutations) showed that bacterial community composition differed significantly by treatment (df = 5, F = 18.42, R^2^ = 0.489, *p* = 0.001), planting time (df = 1, F = 27.28, R^2^ = 0.145, *p* = 0.001) and their interaction (df = 5, F = 9.02, R^2^ = 0.239, *p* = 0.001), together explaining ~87% of the variation in community composition. Multivariate dispersion did not differ significantly among treatments (betadisper, df = 5, F = 2.39, *p* = 0.059), but did differ among the twelve treatments × planting time groups (df = 11, F = 3.17, *p* = 0.002), indicating that part of the treatment × planting time effect may reflect differences in within-group variability rather than purely differences in community centroids. The major bacterial phyla identified were Pseudomonadota, Actinomycetota, Gemmatimonadota, Chloroflexota, Bacteroidota, and Acidobacteriota (Figure 5a). Because composition changed markedly between Day 0 and Day 45 (see Figure 5 and Figure 6), results below are reported for each planting time separately rather than pooled across time.

In the sterile soil inoculated with bioinoculant F2 (T6), Actinomycetota dominated the community immediately after inoculation (Day 0: 80.4% mean relative abundance; genus *Streptomyces* alone: 79.7% of the total community), consistent with direct inoculation of a sterilized, competitor-free soil with *S. gardneri* MC1-2. By Day 45, however, Actinomycetota relative abundance in T6 had fallen to 26.3% (*Streptomyces*: 9.2% of the total community), a range overlapping with several of the non-sterile treatments at the same time point (Figure 5 and Figure 6). This decline most likely reflects a loss of soil sterility over the 45-day incubation, allowing colonization by other taxa, rather than a natural, self-organized restructuring of an existing microbiome, as T6 had no native background community to restructure.

In the non-sterile treatments (T1–T5), bacterial diversity was consistently greater than in T6 at both time points. Pseudomonadota, Gemmatimonadota and Chloroflexota were dominant phyla across these treatments. Pseudomonadota were represented by Alphaproteobacteria and Gammaproteobacteria at the class level; their relative abundance was greatest at Day 45 in the chemically fertilized treatment (T3: 26.8% Actinobacteriota) and in T4 (Actinomycetota 23.1%, with a marked rise in Alphaproteobacteria/*Sphingomonas* and *Methylobacterium–Methylorubrum*, see Figure 6). Actinomycetota was also detected in all non-sterile treatments but generally at lower relative abundance than in T6 at Day 0. In addition, members of the class Bacteroidia (phylum Bacteroidota) were detected in all non-sterile treatments (T1–T5). The class Anaerolineae (phylum Chloroflexota) was notably enriched at Day 45 in T2 and T5 (Figure 5b), consistent with the SMS/bioinoculant amendments in these treatments, and was largely absent from the other treatments.

At the genus level, relative abundance was recalculated as the mean of each sample’s own proportions (rather than pooling raw counts across samples before renormalising) and is reported separately for Day 0 and Day 45 because, as shown in Figure 6, treatment T6 changed markedly between the two time points. Figure 6 shows the top 20 bacterial population classified to genus level, with each Treatment × Time bar renormalized so these 20 genera sum to 100% because genus-level classification resolved only 41.9–52.3% of reads in the non-sterile treatments (T1–T5) and 86.2% of reads in T6, the percentages shown in Figure 6 describe relative proportions among the top 20 genera, not proportions of the whole community (genus abundances expressed as a percentage of the total community, as used elsewhere in this section, are provided in the accompanying relative-abundance workbook). In the non-sterile treatments, the classified community was co-dominated by *Bryobacter*, *Micropepsis* and *Conexibacter* at Day 0, with broadly similar composition across T1–T5. By Day 45, *Sphingomonas* and *Methylobacterium-Methylorubrum* increased markedly in T4 (reaching a combined ~28.6% of the top-20 genera), and *Nocardioides* increased in T6 (17.0% of the top-20 genera at Day 45), illustrating that treatment effects on the genus-level community became more pronounced after 45 days than immediately after treatment application.

In treatment T6, *Streptomyces* sequences accounted for 90.1% of the top-20 genera at Day 0, consistent with direct inoculation of sterile soil with *S. gardneri* MC1-2 and the absence of competing taxa. By Day 45, the *Streptomyces* population had fallen to 25.0% of the top-20 genera in T6, with *Nocardioides*, *Devosia* and *Bacillus* among the genera increasing in its place (Figure 6). As discussed above, we interpret this as most consistent with gradual colonization of the sterile soil over the 45-day incubation rather than an ecological succession driven by competitive restructuring. Across the non-sterile treatments, *Streptomyces* remained comparatively low at both time points in T1–T3 (0.2–3.3% of the top-20 genera) and was highest in T4 at Day 0 (8.8%), followed by T5 at Day 0 (5.8%, bioinoculant F2 application), consistent with direct application of *S. gardneri* MC1-2 (T4) or bioinoculant (T5) into non-sterile soil, followed by a decline by Day 45 in T4 (2.4% of the top-20 genera) and a smaller decline in T5 (5.1%).

## 4. Discussion

Members of the genus *Streptomyces* are increasingly recognized as promising plant growth-promoting microorganisms because of their ability to influence plant growth and development through phytohormone production, nutrient mobilization, siderophore-mediated iron acquisition, stress mitigation, and phytopathogen suppression [31,32,33]. In the present study, the cave-derived actinobacterium *S. gardneri* MC1-2 displayed all plant growth-promoting traits examined, including production of indolic compounds and siderophores and solubilization of tricalcium phosphate, supporting its potential as a candidate for bioinoculant development. Similar multiple plant-beneficial traits have been reported in several Streptomyces strains that subsequently improved plant nutrient acquisition, root development, biomass production, or stress tolerance in various plant species [12,16,34,35,36,37].

The production of total indolic compounds by *S. gardneri* MC1-2 indicates an ability to synthesize IAA-related compounds, although the Salkowski assay does not specifically identify indole-3-acetic acid (IAA). Although IAA was not confirmed by a specific analytical method such as HPLC, LC–MS or GC–MS, microbial production of indolic compounds is widely used as an indicator of auxin-related plant growth-promoting potential, and IAA production is common among beneficial *Streptomyces* species [16,32,35,36,37]. IAA-producing actinobacteria can promote root elongation, lateral root formation, and root hair development, thereby improving water and nutrient uptake [31,35].

Plant growth-promoting actinobacteria can also enhance plant nutrition by increasing the bioavailability of poorly accessible mineral elements, particularly iron and phosphorus. Siderophores are low-molecular-weight, high-affinity Fe^3+^ chelators synthesized by microorganisms to facilitate iron acquisition under iron-limiting conditions and may also improve plant iron uptake [38,39]. *S. gardneri* MC1-2 produced both hydroxamate- and catecholate-type siderophores. Hydroxamate siderophores are particularly common among many actinobacteria, including *Streptomyces*, which produce structurally diverse compounds such as desferrioxamines for iron acquisition and ecological competitiveness [35,36,37,40,41]. The predominance of hydroxamate-type siderophores in *S. gardneri* MC1-2 is consistent with the siderophore profiles frequently reported for members of this genus. Siderophore production may additionally promote plant health by restricting iron availability to competing phytopathogens [42,43].

*S. gardneri* MC1-2 also exhibited an ability to solubilize inorganic phosphate. The decrease in the pH of the culture broth is consistent with organic acid-mediated phosphate solubilization [35,44]. Phosphate-solubilizing *Streptomyces* commonly secrete organic acids and other chelating metabolites that lower the surrounding pH, complex Ca^2+^, and promote solubilization of calcium-bound phosphate compounds [12,45]. Similar phosphate-solubilizing activity has been reported among many actinobacteria, including *Streptomyces* strains that formed clear zones on Pikovskaya’s medium and subsequently enhanced plant growth [35,44].

In addition to its multifunctional plant growth-promoting phenotype, *S. gardneri* MC1-2 was able to grow at a water activity (a_w_) of 0.919 [19], indicating tolerance to reduced water availability under in vitro conditions. This tolerance may be advantageous for bioinoculant development, particularly during formulation and storage, where actinobacterial cells can be exposed to reduced moisture conditions. *Streptomyces* have been reported to combine stress tolerance and plant growth enhancement under adverse environmental conditions [31,32,35,37]. However, the present plant experiment was conducted under non-drought conditions; therefore, the ability of *S. gardneri* MC1-2 to mitigate drought stress in plants requires further investigation. The ability of *S. gardneri* MC1-2 to produce indolic compounds and siderophores, solubilize tricalcium phosphate, and grow at reduced water activity supports its selection as a candidate for bioinoculant development using spent mushroom substrate.

These lignocellulosic materials are attractive as carriers for microbial inoculants due to their high porosity, large internal surface area, and excellent water-retention capacity [9,46]. Cellulose and hemicellulose provide hydrophilic polysaccharide networks supporting moisture retention, whereas lignin contributes structural rigidity and resistance to rapid degradation for the formulated carrier. This combination may provide a physically stable microenvironment beneficial for microbial growth and survival. Mildly acidic pH values of approximately 5.26–5.51 were previously reported for *Pleurotus*-derived SMS, although the final pH depends on the mushroom species, cultivation substrate, and degree of decomposition [46]. The near-neutral pH of the talc-based formulation (F3) was consistent with values commonly found in talc-based microbial inoculants, including streptomycetes, in which CaCO_3_ is added as a buffering agent to adjust carrier pH to approximately 7.0. For example, talc-based formulations of *Streptomyces* spp. CBE, PDK, and MDU were adjusted to pH 7.0 using CaCO_3_ [47]. Similarly, a talc-based formulation of *S. cellulosae* Actino 48 with a pH of 7.0 was developed [48,49]. Therefore, the near-neutral pH observed in F3 most likely resulted from CaCO_3_ rather than from talcum powder.

The relatively stable acidity of the SMS-based formulations may reflect the buffering properties of organic matter within the lignocellulosic matrix [50]. The higher EC values of the SMS-based formulations likely reflect the presence of residual soluble ionic constituents, including nutrients remaining after mushroom cultivation. Spent mushroom substrates from *Pleurotus* cultivation retain considerable amounts of organic matter and mineral nutrients, including N, P, K, Ca, Mg, and trace elements, although their concentrations varied according to the mushroom species and composition of the original cultivation substrate [46,51]. In contrast, talcum powder is a chemically inert mineral carrier with limited available nutrients [2,52]. These physicochemical differences may have important implications for the performance of the formulated bioinoculant and the survival of *S. gardneri* MC1-2 during storage.

This observation suggests that the early increase in EC values resulted primarily from physicochemical processes within the carrier itself rather than the growth of *S. gardneri* MC1-2. Continued weathering and decomposition of SMS have previously been associated with increases in soluble ions such as K^+^, Ca^2+^, Mg^2+^, Na^+^, NO_3_^−^, and Cl^−^ [6,53]. Although metabolic activity of *S. gardneri* MC1-2 cannot be excluded, the absence of any increase in the viable count of this strain suggests that carrier-derived physicochemical processes are the more plausible explanation. Since talcum powder generally contains very low or no organic carbon or nutrients, the formulation may not have maintained an appropriate moisture and nutrient environment required for *S. gardneri* MC1-2 growth. Inorganic carriers primarily function as delivery matrices and often require suitable additives to sustain microbial survival, whereas organic-based carriers can provide microbial inoculants with physical protection, moisture retention, and available nutrients [1]. The rapid loss of *S. gardneri* MC1-2 from F3 therefore suggests poor compatibility between this strain and the talc-based formulation rather than a general inability of talcum powder to support *Streptomyces*. Indeed, some talcum-based formulations have previously been reported to successfully maintain viability of other *Streptomyces* strains for several months, indicating that carrier performance is strain- and formulation-specific [15,25,47,48,49]. Our results and previous literature suggest that, for *S. gardneri* MC1-2, the organic SMS carrier is superior to the nutrient-poor talcum powder.

In addition, although SMS-based formulations F1 and F2 with substantially higher EC values exhibited better survival of *S. gardneri* MC1-2 than F3, the continued increase in EC values did not prevent a reduction in viable populations of *S. gardneri* MC1-2. This observation indicates that a high EC value in itself should not be interpreted as an indicator of superior formulation quality. Since F1 and F2 differed only in the addition of CMC, the differences observed between these formulations may be associated with CMC-induced changes in the physicochemical characteristics of the carrier matrix. CMC is a water-soluble polymer commonly used in microbial formulations to improve cell–carrier adhesion and formulation stability [54,55,56]. The CMC-containing formulation has been demonstrated to support microbial viability and protect microorganisms during storage, although their beneficial effects depend on the microorganisms, carrier composition, and formulation conditions [54,55,56]. Accordingly, the greater increase in EC observed in F2 than in F1 may indicate that incorporation of CMC altered interactions between water and soluble constituents within the SMS matrix. However, the mechanism underlying this difference cannot be established from the present data because moisture content, water activity, ionic composition, and changes in carrier microstructure were not determined.

The survival of *S. gardneri* MC1-2 in SMS-based formulations F1 and F2 probably benefited from both the physical properties and the nutrients of SMS. The comparable persistence of MC1-2 in F1 and F2 therefore supports the suitability of SMS as a carrier matrix under the storage conditions examined, whereas the specific contribution of CMC to F2 cannot be resolved from the present experimental design. Because no CMC-specific control was included and moisture content and water activity were not measured, the observed performance of F2 should be attributed to the combined SMS–CMC formulation rather than specifically to the moisture-retaining properties of CMC. Further studies incorporating CMC controls together with measurements of water activity, moisture retention, ionic composition, carrier microstructure, and cell–carrier interactions would be required to distinguish the contribution of CMC from that of the SMS matrix.

Organic carriers are well known to retain moisture and supply nutrients that support microbial viability during storage [1,3]. SMS, as an organic carrier, differs from nutrient-poor talcum powder in this aspect. This characteristic helps explain why viable *S. gardneri* MC1-2 populations remained detectable for 30 days in the SMS-based formulations. The spore-forming ability of *Streptomyces* may have further benefited its survival since spores are generally resistant to desiccation, nutrient limitation, and temperature stress [13,40].

However, CFU enumeration in this study did not differentiate between viable mycelial fragments and spores. As the formulations may have contained both physiological forms, the observed decline in CFU during storage may partly reflect differences in their survival and persistence within the formulation matrix. Future studies using separately standardized spore and mycelial inocula would enable a more precise assessment of their respective contributions to formulation stability and shelf-life performance.

Our results support spent mushroom substrate as a promising organic carrier for *S. gardneri* MC1-2. Both SMS-based formulations maintained detectable viable populations of *S. gardneri* MC1-2 for at least 30 days across all storage temperatures, whereas viability was completely lost in the talc-based formulation within 7 days. SMS was therefore a more supportive carrier matrix for *S. gardneri* MC1-2 compared to talcum powder. The cellulose-, hemicellulose-, and lignin-rich composition of SMS likely accounts for moisture retention, physical protection, microbial attachment surfaces, and some residual nutrients. However, the considerable reduction in the number of viable *S. gardneri* MC1-2 over time indicates that SMS alone was insufficient to maintain viability over long-term storage. The current SMS-based formulations still need optimization to meet expected requirements for longer-term storage and practical application. Further development should focus on moisture control, CMC concentration, and additional protective agents such as osmoprotectants, antioxidants, or compatible polymer blends capable of maintaining *Streptomyces* spore viability over longer storage periods.

This trend is consistent with previous reports demonstrating that appropriately formulated SMS can serve as a suitable material for horticultural growing media [6,57] and that microbial inoculation can further improve the plant growth-promoting properties of composted SMS [58]. Moreover, plant growth-promoting *Streptomyces* strains have been reported to enhance tomato seed germination and subsequent seedling development [59].

SMS contains residual nutrients and organic matter that can support plant development, but its relatively high EC value may restrict germination when used at inappropriate proportions [6]. This consideration is relevant to the present study because F1 and F2 exhibited substantially higher EC values than the talc-based F3 formulation. Thus, the SMS-based material may provide essential nutrients and a suitable environment for *S. gardneri* MC1-2 survival while imposing some osmotic or physicochemical constraints during the germination stage.

This response should not be interpreted as evidence of an inhibitory effect of *S. gardneri* MC1-2 because direct inoculation of *S. gardneri* MC1-2 in the positive control maintained high germination, while *S. gardneri* MC1-2 also numerically enhanced germination in both SMS-based formulations. This contrasting response suggests that the enhancement effect of *S. gardneri* MC1-2 also depended on carrier compatibility. This interpretation is consistent with the survival experiment, in which *S. gardneri* MC1-2 became undetectable in talc-based F3 within seven days at all temperatures but remained recoverable from both SMS formulations for at least 30 days. These germination and survival data indicate that successful bioinoculant performance depends not only on the intrinsic plant growth-promoting potential of the microbial strain but also on its compatibility with the carrier materials.

The germination results revealed a clear carrier-dependent response. The inoculation with *S. gardneri* MC1-2 increased germination in the SMS-based formulations compared to a reduction in the talc-based formulation. The stimulatory effect of *Streptomyces* on seed germination and early growth is well documented in tomato and other vegetables [10,11]. In a screening of 130 *Streptomyces* strains on four vegetable crops, the bacteria colonized the seed surface and mostly improved radicle and hypocotyl growth yet occasionally caused transient negative effects on germination [60]. In the present study, the SMS-based formulations were associated with higher germination than their corresponding uninoculated carriers, whereas the talc-based formulation showed the opposite trend. These observations suggest that the carrier environment may influence the response to MC1-2 inoculation.

For seedling growth, the SMS-based formulations F1 + *S. gardneri* MC1-2 and F2 + *S. gardneri* MC1-2 produced generally similar fresh weight, dry weight, shoot length, and root length, with overall trends toward higher values than F3 *+ S. gardneri* MC1-2 and the positive control. However, not all pairwise differences were statistically significant, and these results should therefore be interpreted as numerical trends. Similar patterns have been reported for *Streptomyces* inoculation, where increases in radicle and hypocotyl growth of tomato, rocket, and lettuce were observed without consistent statistically significant increases in biomass [60]. Although F1 and F2 showed similar survival patterns during storage, F2 exhibited the numerically highest mean values across the four measured seedling growth parameters. Based on this overall seedling growth profile, F2 was selected for further evaluation in the pot experiment.

However, the seedling growth response should not be attributed solely to the survival of *S. gardneri* MC1-2, because both F1 and F2 maintained viable populations during storage, while uninoculated F2 also produced relatively longer roots (15.97 ± 1.98 cm). These observations suggest that the carrier formulation itself may also have influenced seedling growth. Increasing evidence indicates that spent mushroom substrate (SMS) functions not just as an organic amendment; its lignocellulosic matrix and residual nutrient content enable it to serve as an effective renewable carrier for beneficial microorganisms. For example, recent work demonstrated that SMS outperformed peat and biochar as a carrier for mineral-solubilizing microorganisms, resulting in significantly greater plant biomass and improved soil properties [9]. Thus, the response observed with the SMS-based formulations may reflect the combined influence of the carrier matrix, formulation additives, and the introduced microorganism rather than any single component.

The responses observed with the SMS-based formulations are also consistent with previous studies showing that carrier characteristics can influence the performance of plant growth-promoting *Streptomyces*. For example, *S. fradiae* NKZ-259 produced IAA and significantly promoted tomato growth following formulation in solid carriers [16]. Tomato plants receiving the kaolin-based NKZ-259 formulation nearly doubled tomato root and shoot length, together with a greater fresh and dry biomass than carrier-only treatments. Importantly, the plant growth promotion efficiency of each formulation differed among carrier materials despite supplementation with the same *Streptomyces* strain, indicating that carrier–microorganism compatibility can substantially influence inoculant performance. This carrier-dependent response is comparable with the present study, in which both F1+ *S. gardneri* MC1-2 and F2 + *S. gardneri* MC1-2 generally promoted better seedling growth than the talc-based F3 + *S. gardneri* MC1-2 formulation.

This growth promotion is consistent with the well-recognized role of *Streptomyces* as a plant growth-promoting bacterium that stimulates germination and seedling development through indole-3-acetic acid production, phosphate solubilization, and siderophore production, together with efficient rhizosphere colonization [10,11]. *S. gardneri* MC1-2 also exhibited in vitro traits commonly associated with plant-beneficial activity, in particular total indolic compounds, siderophore production and phosphate solubilization. These functions are widely reported among plant-beneficial *Streptomyces* and provide complementary mechanisms through which inoculation may influence early plant development.

The comparison between F2 with and without *S. gardneri* MC1-2 is also informative. Although uninoculated F2 produced relatively long roots, the addition of *S. gardneri* MC1-2 increased mean fresh weight from 0.727 to 1.510 g, dry weight from 0.107 to 0.183 g, shoot length from 4.03 to 6.10 cm, and root length from 15.97 to 18.13 cm. These results suggest that the favorable response of F2 + *S. gardneri* MC1-2 was not attributable solely to the nutritional properties of SMS. The organic SMS carrier and the introduced microorganism appear to have at least partially exerted complementary effects, as these pairwise differences were not statistically significant.

The survival experiment provides additional supporting evidence for an influence of the carrier materials. *S. gardneri* MC1-2 remained detectable at 10^4^ CFU/g in both SMS-based formulations after 30 days of storage, whereas viable cells were completely lost in the talc-based F3 formulation within 7 days at all tested temperatures. Thus, the inferior seedling response associated with F3 + *S. gardneri* MC1-2 may partly reflect poor compatibility between *S. gardneri* MC1-2 and the talc-based formulation. Previous formulation studies reported that the effectiveness of *Streptomyces* inoculants depends on growth-promoting properties of the strain and the carrier used [16].

The superior seedling growth after direct inoculation with *S. gardneri* MC1-2 fits with the observed growth-promoting traits of this strain, namely indolic compound production, phosphate solubilization, and siderophore production. These properties are known to improve nutrient uptake, promote root development, and support plant growth [12,32]. Similarly, other *Streptomyces* species were reported to enhance tomato biomass and shoot development via phytohormone production and improved nutrient availability [16,31].

In the pot experiment, the chemical fertilizer treatment served as the positive agronomic benchmark, giving the greatest shoot length, chlorophyll and carotenoid content, and TSS of all treatments, reflecting the ready availability of NPK. Direct inoculation with *S. gardneri* MC1-2 gave fresh weight, dry weight, and shoot length that were statistically comparable to T3 under the conditions tested, which does not imply biological equivalence to synthetic fertilizer; this response over 45 days is nevertheless in line with the plant growth-promoting mechanisms reported for actinobacteria such as IAA production, phosphate solubilization, and siderophore production [12,16]. In contrast, the two treatments containing SMS in non-sterile soil produced the lowest biomass, TSS, and chlorophyll content. Because chlorophyll and accessory pigments such as carotenoids are essential for photosynthesis [61], the low pigment content offers one explanation for the low TSS. SMS is rich in organic carbon that is not fully decomposed, and its high carbon-to-nitrogen ratio can temporarily immobilize soil nitrogen and limit plant growth [62]. This may explain why our SMS treatments underperformed relative to earlier studies in which SMS showed growth enhancement. For example, in strawberry, the addition of fresh SMS as a peat substitute significantly affected yield, leaf area, and both fresh and dry weight [63]. Similarly, PGPR-treated SMS improved growth [64]. This discrepancy may result from differences in microbial species, growth stage, and the role assigned to SMS in those studies. SMS was generally used as a major, often extensively decomposed substrate component, whereas here it served mainly as a minor, less-decomposed carrier.

SMS is a lignocellulosic material composed primarily of cellulose, hemicellulose, and lignin and undergoes gradual microbial decomposition after soil application, with its degradation behavior influenced by substrate composition and soil microbial activity [53]. This natural degradation differs from engineered encapsulation systems, in which polysaccharide-based matrices can protect microbial cells and provide controlled or gradual microbial release into soil [55]. The relationship between SMS degradation and the persistence or release of *S. gardneri* MC1-2 merits further investigation.

CMC is commonly used in microbial formulations as a thickening and suspending agent and is generally considered biocompatible; however, its effects on plant responses can depend on concentration and application conditions [65,66]. Excessive CMC may adversely affect plant growth by altering the physical environment around roots and influencing water, nutrient, and oxygen movement [66]. In the present pot experiment, F2 contained 1% (*w*/*w*) CMC, and 5 g of F2 was applied to 50 g of peat moss, corresponding to approximately 0.05 g CMC per pot or 0.09% (*w*/*w*) of the formulation–peat moss mixture. Given its low inclusion level, CMC constituted only a minor component of the applied treatment; however, the influence of CMC concentration on plant responses warrants further investigation.

These results suggested that neither *S. gardneri* MC1-2 nor the SMS carrier imposed measurable osmotic or ionic stress despite the higher EC values of the SMS-based treatments. *Streptomyces* root colonization is reported to be strain-specific, and colonization efficiency depends on the inoculation method, with better results obtained from direct substrate inoculation than from seed coating [67].

Distinct responses were also associated with the SMS carrier. Bacteroidota, represented mainly by the class Bacteroidia, occurred in all non-sterile treatments and was relatively more abundant in the SMS-amended soils (T2 and T5). Members of this bacterial group are common in compost-amended soils and well known for their extensive carbohydrate-active enzyme systems that degrade the cellulose and hemicellulose of lignocellulosic residues [68,69]. Similarly, Anaerolineae, within the phylum Chloroflexota, were detected only in the SMS-supplemented treatments (T2 and T5). Their restriction to these treatments most likely reflects microenvironments within the lignocellulosic carrier rather than anaerobic conditions in the bulk soil. Anaerolineae are typically associated with anaerobic microhabitats and have been reported in the salt-marsh rhizosphere [70]. A relatively high abundance of Pseudomonadota, particularly Alphaproteobacteria and Gammaproteobacteria, was observed in the chemically fertilized treatment (T3). These taxa are metabolically versatile, fast-growing bacteria that respond rapidly to nutrient enrichment, and their greatest abundance in the chemical fertilizer treatment (T3) is consistent with its higher nutrient availability [71,72]. The decomposed carrier detection across all non-sterile treatments further reflects the stability of the native soil microbial community.

The bacterial community analysis also helps explain the tomato growth responses observed in the pot experiment. *Actinomycetes* strongly dominated T6 immediately after inoculation of the sterile soil (Day 0: 80.4% relative abundance), and although this dominance declined by Day 45 (26.3%), tomato growth was statistically comparable to that observed following direct inoculation with *S. gardneri* MC1-2 (T4) and chemical fertilizer (T3). This finding suggests that successful establishment of *S. gardneri* MC1-2 was sufficient to support tomato growth in sterile soil. The decline in relative abundance between Day 0 and Day 45 is consistent with reports that soil bioinoculants, including *Streptomyces*-based biocontrol strains, typically show limited long-term persistence in soil once they must compete for resources and niche space [73], and with the rapid recolonization dynamics reported for sterile soils during incubation, in which newly introduced microorganisms can be diluted or replaced by other microorganisms within weeks [74]. The high relative abundance of the introduced *S. gardneri* MC1-2 indicates robust growth in the absence of native microbial populations rather than superior adaptive fitness within a complex soil ecosystem. Rapid growth in this sterile environment therefore does not demonstrate an ability of the introduced taxon to outcompete or restructure the native population. The lower population of *S. gardneri* MC1-2 and the reduced plant biomass observed in T5 suggest that competition from native microorganisms rather than carrier performance likely limited inoculant establishment and its efficacy in non-sterile soil. These findings emphasize that the success of microbial bioinoculants depends not only on the physiological properties of the inoculated strain or the quality of the carrier material but also on their interactions with native soil microbial communities [75].

Bacterial community analysis and re-isolation results consistently show that the SMS-based formulation F2 supports the establishment of *S. gardneri* MC1-2, but that its performance depends on the resident native bacterial community. In sterile soil without microbial competition, the inoculated actinobacterium became the dominant bacterial group and maintained plant growth statistically comparable to that of the chemical fertilizer treatment. However, the effect of nutrients released during the soil sterilization process cannot be ruled out. Therefore, the observed superior establishment of *S. gardneri* MC1-2 in T6 cannot be attributed solely to the absence of microbial competition. In contrast, under non-sterile conditions, the native bacterial community limited inoculant establishment. This observation highlights the need for formulation improvements that enhance microbial competitiveness and persistence in natural soils, which remains an important challenge for successful field application.

Several limitations should be considered when interpreting these results. The pot trial lasted 45 days and assessed vegetative growth parameters only, so no conclusion can be drawn regarding flowering, fruit set, yield, or performance over a full crop cycle. In addition, the chemical fertilizer treatment (T3) remained the strongest treatment overall, giving the highest total chlorophyll and carotenoid contents consistent with its highest total soluble sugar content, which reflects the ready availability of NPK. A further limitation is the absence of carrier-additive controls in the pot experiment. CMC was incorporated at only 1% (*w*/*w*) of the SMS carrier as a formulation adjuvant to improve adhesion and moisture retention, and thereby to maintain the viability of *S. gardneri* MC1-2 during storage, rather than as a plant nutrient or growth-promoting amendment. Given this low application rate and the absence of a reported direct plant-growth effect of CMC at this dose, a CMC-only soil amendment was not expected to contribute measurably to plant performance and was therefore not included as a separate treatment. The talc-based formulation (F3) failed to maintain the viability of *S. gardneri* MC1-2 during storage and was consequently not carried forward to the pot experiment, so a talc-only soil amendment was not relevant to the treatments finally evaluated. The individual contribution of the carriers was nevertheless partly addressed at the seedling stage, where each formulation (F1, F2 and F3) was tested with and without *S. gardneri* MC1-2 (Table 6); formulations supplemented with the strain gave more favorable growth trends than the corresponding carrier-only formulations, indicating that the observed growth benefit was associated with the inoculated strain rather than with the carrier matrix. Further greenhouse-scale studies over a longer growing period are therefore still required. A further consideration is that T4, T5 and T6 received unequal inoculum doses since bioinoculant performance can be influenced by both formulation characteristics and the number of viable cells delivered to the plant–soil system [1]. T4, T5, and T6 received approximately 10^6^, 10^7^, and 10^7^ CFU per pot, respectively. Thus, T4 and T5 differed not only in the mode of inoculum delivery but also by approximately one order of magnitude in the initial viable-cell number. Consequently, the observed differences in plant responses between these treatments cannot be attributed specifically to the delivery format, as the effects of formulation and inoculum dose cannot be separated with the present design. Although T4 and T5 can be considered independent treatments representing direct inoculation and F2 application, respectively, their comparison does not provide a dose-controlled assessment of the efficacy of these delivery approaches. Accordingly, no conclusion is drawn regarding whether direct application of MC1-2 or its delivery through the F2 formulation is more effective. Future experiments using equivalent viable-cell number for each delivery method, will be necessary to distinguish formulation effects from dose-dependent responses.

## 5. Conclusions

In summary, this study shows that spent mushroom substrate (SMS) can serve as an organic carrier for the plant growth-promoting actinobacterium *S. gardneri* MC1-2. Both SMS-based formulations maintained viable populations of S. gardneri MC1-2 throughout 30 days of storage at 4 °C, 30 °C and 40 °C, although viability declined by approximately 3 log CFU, whereas the conventional talc-based carrier lost viability completely within the first 7 days. This observation suggests that the lignocellulosic SMS is a better material for supporting microbial survival during storage. The SMS-based formulation F2, supplemented with 1% (*w*/*w*) CMC, provided satisfactory survival during storage at high temperature together with superior tomato seedling growth.

Direct inoculation with *S. gardneri* MC1-2 clearly promoted tomato growth. The SMS based formulation F2 gave similar growth in sterile soil, where the strain established and became the dominant bacterial group in both the re-isolation and the community sequencing data. In non-sterile soil, the SMS based formulation F2 performed less well, with lower recoverable populations of *S. gardneri* MC1-2 and a more diverse native bacterial community, which points to a possible competition between the native bacteria and the introduced strain as a factor limiting establishment in natural soil. Our findings highlight that the efficacy of a bioinoculant depends not only on the carrier and the strain but also on the interactions between the introduced organism and the native soil microbial community. However, the SMS-based formulation F2 showed potential for use in systems with limited competition from native microorganisms, such as sterilized substrates or soilless systems. Successful application in non-sterile agricultural soils will require further formulation optimization, improved inoculant stability, and validation under field conditions.

## Figures and Tables

**Table 1:** Details of experimental treatments.

Treatment	Substrate (g)				Supplements (mL)	
	Non-Sterile Soil	Sterile Soil	SMS	Bioinoculant F2	Spore Suspension (2.43 × 10^6^ CFU/mL)	Chemical Fertilizer
T1	55	-	-	-	-	-
T2	50	-	5	-	-	-
T3	55	-	-	-	-	6
T4	55	-	-	-	6	-
T5	50	-	-	5	-	-
T6	-	50	-	5	-	-

T1, non-sterile soil control; T2, non-sterile soil + SMS; T3, non-sterile soil + chemical fertilizer (16:16:16); T4, non-sterile soil + spore suspension of *S. gardneri* MC1-2; T5, non-sterile soil + bioinoculant F2; T6, sterile soil + bioinoculant F2. SMS, spent mushroom substrate; F2, SMS supplemented with 1% (*w*/*w*) carboxymethyl cellulose (CMC) and inoculated with *S. gardneri* MC1-2; CFU, colony-forming units; -, not applied.

**Table 2:** Physicochemical Properties of Bioinoculant Formulations Including pH, Electrical Conductivity (EC), and Viable Cell Count of *S. gardneri* MC1-2.

Bioinoculant Formulation	pH	EC (μS/cm)	Number of *S. gardneri* MC1-2 (CFU/g)
F1	5.85	2263	5.35 ± 1.48 × 10^7^
F2	5.96	2290	3.90 ± 1.13 × 10^7^
F3	6.94	427	3.70 ± 0.42 × 10^7^

F1, spent mushroom substrate (SMS); F2, SMS supplemented with 1% (*w*/*w*) carboxymethyl cellulose (CMC); F3, talcum powder supplemented with 1% (*w*/*w*) CMC and 1.5% (*w*/*w*) CaCO_3_; EC, electrical conductivity; CFU, colony-forming units. Viable counts are presented as mean ± SD (*n* = 3).

**Table 3:** pH of bioinoculant formulation at 4 °C, 30 °C, and 40 °C for 7, 15, and 30 days.

Bioinoculant Formulation	pH									
	Day 0	Day 7			Day 15			Day 30		
		4 °C	30 °C	40 °C	4 °C	30 °C	40 °C	4 °C	30 °C	40 °C
F1	5.85	6.24	6.17	6.14	6.27	6.23	6.16	5.93	5.92	5.96
F2	5.96	6.16	6.16	6.19	6.18	6.28	6.38	5.98	6.01	5.99
F3	6.94	7.85	7.85	7.95	8.82	9.12	9.10	7.70	8.12	8.12

F1, spent mushroom substrate (SMS); F2, SMS supplemented with 1% (*w*/*w*) carboxymethyl cellulose (CMC); F3, talcum powder supplemented with 1% (*w*/*w*) CMC and 1.5% (*w*/*w*) CaCO_3_.

**Table 4:** EC value of bioinoculant formulation at 4 °C, 30 °C, and 40 °C for 7, 15, and 30 days.

Bioinoculant Formulation	EC Value (μS/cm)									
	Day 0	Day 7			Day 15			Day 30		
		4 °C	30 °C	40 °C	4 °C	30 °C	40 °C	4 °C	30 °C	40 °C
F1	2263	2970	3010	3020	3030	3010	2790	2790	2810	2720
F2	2290	3040	3150	3200	3040	3070	2900	3100	2990	3350
F3	427	316	288	279	332	269	265	341	336	321

F1, spent mushroom substrate (SMS); F2, SMS supplemented with 1% (*w*/*w*) carboxymethyl cellulose (CMC); F3, talcum powder supplemented with 1% (*w*/*w*) CMC and 1.5% (*w*/*w*) CaCO_3_; EC, electrical conductivity.

**Table 5:** Viable counts of *S. gardneri* MC1-2 (CFU/g, mean ± SD, n = 3) in the three bioinoculant formulations during storage at 4, 30, and 40 °C for 0, 7, 15, and 30 days.

Formulation	Storage Temperature	Day 0	Day 7	Day 15	Day 30
F1 (SMS)	4 °C	5.35 ± 1.48 × 10^7^	3.70 ± 1.85 × 10^7^	3.23 ± 0.84 × 10^6^	3.84 ± 0.70 × 10^5^
	30 °C	5.35 ± 1.48 × 10^7^	4.60 ± 0.10 × 10^7^	3.47 ± 0.57 × 10^6^	2.67 ± 1.16 × 10^4^
	40 °C	5.35 ± 1.48 × 10^7^	4.10 ± 0.53 × 10^7^	4.40 ± 0.13 × 10^6^	3.70 ± 0.70 × 10^4^
F2 (SMS + CMC)	4 °C	3.90 ± 1.13 × 10^7^	3.50 ± 0.28 × 10^7^	3.10 ± 1.13 × 10^5^	8.60 ± 0.49 × 10^4^
	30 °C	3.90 ± 1.13 × 10^7^	2.15 ± 0.49 × 10^7^	6.47 ± 0.51 × 10^5^	5.03 ± 1.04 × 10^4^
	40 °C	3.90 ± 1.13 × 10^7^	3.20 ± 0.00 × 10^7^	5.10 ± 0.44 × 10^6^	5.30 ± 0.50 × 10^4^
F3 (Talcum + CMC + CaCO_3_)	4 °C	3.70 ± 0.42 × 10^7^	ND	ND	ND
	30 °C	3.70 ± 0.42 × 10^7^	ND	ND	ND
	40 °C	3.70 ± 0.42 × 10^7^	ND	ND	ND

F1, spent mushroom substrate (SMS); F2, SMS supplemented with 1% (*w*/*w*) carboxymethyl cellulose (CMC); F3, talcum powder supplemented with 1% (*w*/*w*) CMC and 1.5% (*w*/*w*) CaCO_3_; SMS, spent mushroom substrate; CMC, carboxymethyl cellulose; ND, non-detected.

**Table 6:** Growth of tomato seedlings grown in bioinoculant formulations with and without *S. gardneri* MC1-2 supplement after 21 days of cultivation.

Treatment		Fresh Weight (g)	Dry Weight (g)	Shoot Length (cm)	Root Length (cm)
With *S. gardneri* MC1-2	F1	1.477 ± 0.981 ^a^	0.170 ± 0.085 ^ab^	5.93 ± 1.16 ^b^	14.57 ± 0.78 ^bc^
	F2	1.510 ± 0.165 ^a^	0.183 ± 0.012 ^b^	6.10 ± 0.17 ^b^	18.13 ± 1.96 ^c^
	F3	1.187 ± 0.863 ^a^	0.153 ± 0.067 ^ab^	5.63 ± 1.33 ^ab^	14.63 ± 4.58 ^bc^
	Positive Control	0.727 ± 0.232 ^a^	0.080 ± 0.017 ^a^	4.73 ± 0.64 ^ab^	7.87 ± 1.19 ^a^
Without *S. gardneri* MC1-2	F1	0.997 ± 0.417 ^a^	0.133 ± 0.059 ^ab^	4.97 ± 0.93 ^ab^	13.03 ± 0.50 ^b^
	F2	0.727 ± 0.366 ^a^	0.107 ± 0.012 ^ab^	4.03 ± 0.67 ^a^	15.97 ± 1.98 ^bc^
	F3	1.277 ± 0.309 ^a^	0.153 ± 0.031 ^ab^	5.57 ± 0.74 ^ab^	13.10 ± 1.15 ^b^
	Negative Control	1.280 ± 0.546 ^a^	0.153 ± 0.064 ^ab^	5.33 ± 0.91 ^ab^	12.83 ± 1.63 ^b^

data represent mean values of three replicates ± SD. The different superscript letters (a, b, c, ab, bc) within the same column indicate significant differences between treatments according to Tukey’s multiple range test, *p* < 0.05.

## Data Availability

The data supporting the findings of this study are available from the corresponding author upon reasonable request.

## Abbreviations

The following abbreviations are used in this manuscript:

ANOVA	analysis of variance
CAS	Chrome Azurol S
CMC	carboxymethyl cellulose
DADA2	amplicon denoising pipeline
EC	electrical conductivity
IAA	indole-3-acetic acid
ISP2	International *Streptomyces* Project medium 2
MSB	mannitol soybean agar
ND	not detected
NMDS	non-metric multidimensional scaling
NPK	nitrogen–phosphorus–potassium (chemical fertilizer)
PCR	polymerase chain reaction
PERMANOVA	permutational multivariate analysis of variance
PGP	plant growth-promoting
PGPR	plant growth-promoting rhizobacteria
PVK	Pikovskaya’s agar
SD	standard deviation
SMS	spent mushroom substrate
TSS	total soluble sugar
